# Alternating Hemiplegia of Childhood: gastrointestinal manifestations and correlation with neurological impairments

**DOI:** 10.1186/s13023-020-01474-w

**Published:** 2020-09-03

**Authors:** Milton Pratt, Julie Uchitel, Nancy McGreal, Kelly Gordon, Lyndsey Prange, Melissa McLean, Richard J. Noel, Blaire Rikard, Mary K. Rogers Boruta, Mohamad A. Mikati

**Affiliations:** 1 Division of Pediatric Neurology and Developmental Medicine, Duke University Health System, 2301 Erwin Rd., Durham, NC 27710 USA; 2 Divison of Gastroenterology, Department of Pediatrics, Duke University, Durham, NC USA; 3 Department of Speech Pathology and Audiology, Duke University Health System, Durham, NC USA

**Keywords:** Alternating hemiplegia of childhood, *ATP1A3*, Non-paroxysmal disability index, GMFCS

## Abstract

**Background:**

Alternating Hemiplegia of Childhood (AHC) is caused by mutations of the ATP1A3 gene which is expressed in brain areas that include structures controling autonomic, gastrointestinal, gut motility and GABAergic functions. We aimed to investigate, in a cohort of 44 consecutive AHC patients, two hypotheses: 1) AHC patients frequently manifest gastrointestinal, particularly motility, problems. 2) These problems are often severe and their severity correlates with neurological impairments.

**Results:**

41/44 (93%) exhibited gastrointestinal symptoms requiring medical attention. For these 41 patients, symptoms included constipation (66%), swallowing problems (63%), vomiting (63%), anorexia (46%), diarrhea (44%), nausea (37%), and abdominal pain (22%). Symptoms indicative of dysmotility occurred in 33 (80%). The most common diagnoses were oropharyngeal dysphagia (63%) and gastroesophageal reflux (63%). 16 (39%) required gastrostomy and two fundoplication. Severity of gastrointestinal symptoms correlated with non-paroxysmal neurological disability index, Gross Motor Function Classification System scores, and with the presence/absence of non-gastrointestinal autonomic dysfunction (*p* = 0.031, 0.043, Spearman correlations and 0.0166 Cramer’s V, respectively) but not with the paroxysmal disability index (*p* = 0.408).

**Conclusions:**

Most AHC patients have gastrointestinal problems. These are usually severe, most commonly are indicative of dysmotility, often require surgical therapies, and their severity correlates with that of non-paroxysmal CNS manifestations. Our findings should help in management-anticipatory guidance of AHC patients. Furthermore, they are consistent with current understandings of the pathophysiology of AHC and of gastrointestinal dysmotility, both of which involve autonomic and GABAergic dysfunction.

## Introduction

Alternating Hemiplegia of Childhood (AHC) is a complex neurodevelopmental disorder. Mutations in the alpha 3 subunit of the sodium potassium ATPase are the cause of AHC in approximately 75% of patients. In some of the remaining patients the cause is *ATP1A2* mutations, while in the rest, the etiology is still unknown. AHC is diagnosed according to six clinical criteria, known as the Aicardi criteria: (1) onset prior to 18 months of age; (2) paroxysmal hemiplegia episodes; (3) bilateral hemiplegia, or quadriplegia episodes; (4) other paroxysmal manifestations, such as abnormal eye movements, nystagmus, strabismus, ataxia, dystonia, choreoathetosis, tonic spells, or autonomic disturbances; (5) evidence of permanent neurological dysfunction, which can manifest as cognitive impairment, developmental delay, and/or persistent motor deficits such as spastic diplegia/quadriplegia, hypotonia, ataxia, choreoathetosis, or dystonia; (6) sleep relieves symptoms, although attacks may resume soon after awakening [1–3]. Although episodes of autonomic dysfunction, such as sweating and flushing, are known to occur in AHC, gastrointestinal symptoms have not previously been recognized to be an important problem in this disorder. Additionally, despite an expanding literature on the syndrome, there are not studies, to our knowledge, of AHC related gastrointestinal problems, their extent, prognosis, response to therapy, complications, etiology or association with other AHC manifestations [4–7]. Interestingly, however, we have commonly observed gastrointestinal problems in AHC patients being followed in our Duke Multidisciplinary AHC Clinic. In addition, *ATP1A3* is highly expressed in brain areas that control the autonomic nervous system and consequently gastrointestinal motility, such as the hypothalamic and vagus nerve nuclei [8, 9]. This gene is also expressed in motor brain stem nuclei that control swallowing and in GABAergic interneurons that can modulate motility [10]. These findings support the possibility that gastrointestinal symptoms may be prominent manifestations of AHC. Thus, based on the above observations, we generated the following two hypotheses. First, patients with AHC frequently manifest gastrointestinal problems, particularly those related to dysmotility. Second, these gastrointestinal symptoms are often prominent and correlate with the severity of the other AHC neurological manifestations.

## Methods

Retrospective review of our prospective database of a cohort of 44 consecutive patients, seen over a period of 4.5 years, with AHC. These patients were seen for their clinical care at the Duke AHC Multidisciplinary Clinic over a 1 year period. All 44 patients fulfilled the aforementioned Aicardi criteria. Families provided informed consent to participate in the study. Patient data were entered into our Institutional Review Board approved database and then analyzed.

### Symptoms

The frequency of gastrointestinal symptoms requiring medical attention was recorded. We then determined the mean and median number of symptoms per patient, as well as the range. For each patient, gastrointestinal symptom severity was scored on an ordinal scale of 0 to 3. Absence of symptoms was scored as “0,” symptoms that warranted medical attention but did not require intervention were scored as “1,” symptoms that required medication interventions were scored as “2,” and symptoms that required surgical intervention were scored as “3.” If a patient had more than one symptom, then the score of the most severe symptom was used in the correlation analyses.

### Diagnoses, procedures, interventions

Gastrointestinal diagnoses were made clinically and, when clinically indicated, were confirmed by endoscopy and radiological procedures. Interventions (categorized as either medical or surgical) were also noted. Specific interventions included dietary advice, medication management, gastrostomy tube (G-tube) placements, and Nissen fundoplication.

### Correlations with paroxysmal and non-paroxysmal AHC disease manifestations

Using the Spearman rank order correlation, gastrointestinal symptom severity was correlated with previously established and published measures of AHC neurological disease severity scores: paroxysmal disability index scores, non-paroxysmal disability index scores, and Gross Motor Function Classification System (GMFCS) scores [11, 12]. Correlation coefficients with respective *p*-values were calculated.

The paroxysmal disability index (range 0–24) and the non-paroxysmal disability index (0–15) were calculated based on previously described methods for patients with AHC [11]. The paroxysmal disability index was based on the three variables that determined the severity of plegic and dystonic attacks: (i) severity; (ii) frequency and (iii) duration. It was defined in our study as the sum of scores allocated to the three variables. The three variables were determined for both plegic and tonic/dystonic attacks (maximum of six variables/patient) and were scored as follows: (i) severity, number of extremities involved (one limb = 1 point, more than one limb = 2 points, both sides or 4 limbs = 3 points); (ii) frequency (< 1 attack/year = 1 point, monthly attacks = 2 points, weekly = 3 points, daily = 4 points); and (iii) duration (< 1 h = 1 point, 1–6 h = 2 points, 6–12 h = 3 points, 12–24 h = 4 points,> 24 h = 5 points).

The non-paroxysmal disability index (range 0–15) calculated in our study is a slight modification of the index used in the article by Panagiotakaki [11]. It consisted of the sum of scores allocated to seven variables that determined the severity of global neurological imapriment: (i) the ability to walk independently (independent walking = 0 points, walking with help = 1 point, not possible = 2 points); (ii) behavioral disorder (no = 0 points, yes = 1 point); (iii) communication disorder (no = 0 points, yes = 1 point); (iv) gross motor abnormalities (as determined by their GMFCS score: 0 = 0 points, I = 1 point; II,IIIm = 2 points; IV,V = 3 points); (v) fine motor abnormalities as determined by the Manual Ability Classification System (1 = 0 points, 2 = 1 points, 3,4 = 2 points, 5 = 3 points) [12]; (vi) movement disorders (0 = absent, 1 = present; include chorea, dystonia, myoclonus, tremor and complex movement disorders); and (vii) cognitive impairment [variables (iv–vii) were quantified as follows: none = 0 points, mild = 1 point, moderate = 2 points, severe = 3 points, profound =4 points].

The GMFCS is a classification system traditionally used by clinicians and researchers to assess the everyday gross motor function of children and adults with cerebral palsy, and more recently, AHC [12]. Each level in this classification system incorporates a specific age range and age-specific skills that corresponded to the following: (I) walks without limitations; (II) walks with limitations; (III) walks using a hand-held mobility device; (IV) self-mobility with limitations – may use powered mobility; (V) transported in a manual wheelchair.

Lastly the concordance between gastrointestinal symptoms (present/absent) and non-gastrointestinal type autonomic dysfunction (present/absent), such as flushing, sweating or tachycardia, was calculated via the Cramer’s V, a statistic used to measure the strength of association between two nominal variables.

## Results

### Patient characteristics

The mean age of our patients was 10.41 + 9.48 years (range 3–45, 18 males, 26 females). All fulfilled the AHC clinical criteria. Thirty of those tested (71.4%) had sodium potassium ATPase mutations, 12 (28.6%) had no mutations, and 2 were not tested. Mutations included D801N (8), E815K (5), G89D (3), G755C (2) and other mutations each seen in a single patient. Mean (+ SD) GMFCS was 1.77 + 0.95, paroxysmal disability index was 12.86 + 4.68 and non-paroxysmal disability index was 5.39 + 3.08. 29/44 of our patients had normal MRI and 14/44 had an abnormal one. 1/44 had an MRI conducted elsewhere, and the result in that patient was not retrievable. Notable MRI findings included: signal abnormalities in the white matter tracts (4), cerebellar atrophy (4), signal abnormalities in the left hippocampus (1), nonspecific T2 foci prolongation (1), mild diffuse cortical atrophy (1), right occipital polymicrogyria (1), prominent subarachnoid spaces (1), and frontoparietal atrophy (1). The mean weight percentile and standard deviation were 34.4+ 31.1.

### Symptoms (Table 1)

**Fig. 1:**
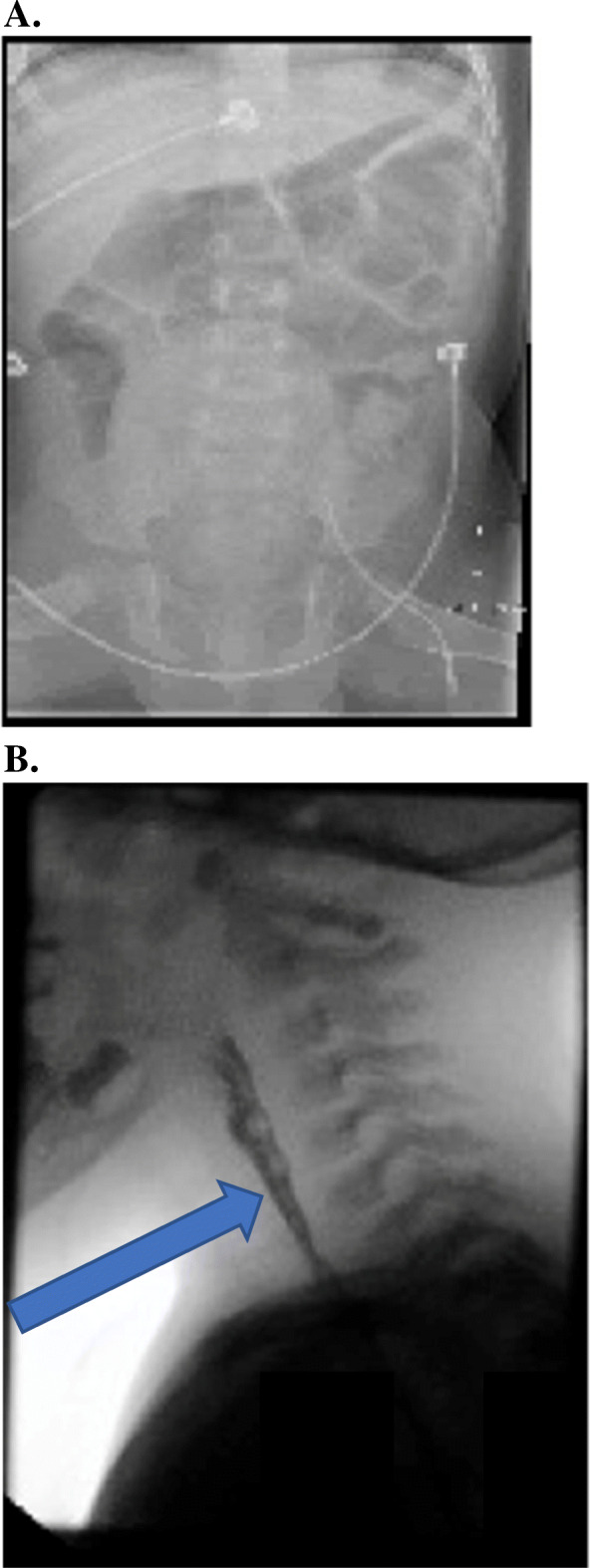
**a**. Three year old male with a de novo *ATP1A3* mutation (V589F). There is gaseous distension of the bowel noted at baseline, suggestive of dysmotility. **b**. Three year old female with a de novo *ATP1A3* mutation (L839P). Videofluoroscopic swallow study demonstrates evidence of orophyarngeal dysfunction complicated by post-swallow residue collection in the pharynx (blue arrow)

Forty-one out of 44 patients (93%) exhibited gastrointestinal symptoms that warranted medical attention. In all 41 patients, these symptoms occurred independently of the hemiplegia, dystonia, seizure or other AHC type spells. Also, often, gastrointestinal symptoms occurred in association with such spells too. The mean number of gastrointestinal symptoms per patient was 3.84 (median 4, range 0–9). Average gastrointestinal symptom severity score was 1.97 (median 2 range 1–3). Constipation with distention (Fig. 1a) was more common than diarrhea but many patients alternated between the two. Constipation was observed in 27 (66%), diarrhea in 18 (44%), with 12 (29%) manifesting both. Swallowing difficulty was observed in 26 (63%, Fig. 1b). Twenty six patients (63%) presented with recurrent vomiting. The above symptoms resulted in recurrent aspiration episodes that were observed in 10 patients (24%). Nineteen patients (46%) developed failure to thrive due to anorexia and other gastrointestinal symptoms, and this commonly was associated with worsening of the AHC paroxysmal manifestations of hemiplegia, dystonia and seizures. 11 (27%) presented with dehydration secondary to gastrointestinal symptoms not related to intercurrent infections. In 5 of the 11, dehydration was due to anorexia, in 4 due to diarrhea, and in 2 due to vomiting. Of the MRI positive patients, 13/14 had gastrointestinal symptoms and 28/30 of the MRI negative patients had such symptoms. Gastrointestinal symptoms requiring intervention occurred in 9/14 MRI positive patients and in 22/30 of MRI negative patients. Neither of these comparisons achieved statistical significance (*p* = 1.0 and 0.72 respectively). We observed gastrointestinal symptoms in both groups of mutation positive and mutation negative patients. Of the mutation positive patients, 27/30 had gastrointestinal symptoms and 12/12 of the mutation negative patients had these symptoms. Gastrointestinal symptoms requiring intervention occurred in 18/30 mutation positive patients and in 11/12 of mutation negative patients. Neither of these comparisons achieved statistical significance (*p* = 0.54 and 0.07 respectively). We compared the severity of gastrointestinal symptoms in patients with high disability index scores (above the median or at it) as compared to those with low disability index scores (below the median). We found, as expected, that more patients with the high nonparoxysmal disability index needed intervention for their gastrointestinal symptoms (79%) than those with high paroxysmal disability index (68%). Similarly, less patients with lower nonparoxysmal disability index required intervention (60%) than those with low paroxysmal disability index (74%). These data are consistent with our correlation analysis that showed that higher gastrointestinal symptom severity correlated with higher nonparoxysmal disability index scores but not with the paroxysmal disability index. In order to probe the potential effects of different medications in our patients, we categorized the medications into different classes and determined the numbers of patients with gastrointestinal symptoms in each category. We found the following numbers of patients had gastrointestinal symptoms: 34 of the 36 patients who were on benzodiazepines, 24/26 patients who were on other antiepileptics, 30/33 patients who were on flunarizine and 14/15 patients who were on anticholinergic medications.

**Table 1:** Gastrointestinal symptoms and procedures in our cohort

**Symptoms**	
**Upper Gastrointestinal Symptoms**	**Number of Patients Per Symptom**
Dysphagia	26
Vomiting	26
Weight Loss/Failure to Thrive/Anorexia	19
Nausea	15
Sialorrhea	8
**Lower Gastrointestinal Symptoms**	**Number of Patients Per Symptom**
Constipation	27^**a**^
Diarrhea	18^**a**^
Abdominal Pain	9^b^
**Procedures**	
**Type of Procedure**	**Number of Patients with Abnormal Results/Total Number of Patients Undergoing the Test (% of patients)**
Video Fluoroscopic Swallow Study	10/10 (100%)
Upper Endoscopy	2/7 (28.5%)
Nuclear Medicine Gastric Emptying Study	4/5 (80%)
Upper GI X-Ray Series	0/4 (0%)
Abdominal Ultrasound	0/2 (0%)
Colonoscopy w/ Biopsy	0/2 (0%)
Upright Modified Barium Swallow Study	1/1 (100%)
CT Abdomen	1/1 (100%)^c^
Anorectal Manometry	0/1 (0%)

^a^33/41 patients with gastrointestinal symptoms exhibited either constipation or diarrhea. Of these 33 patients, 12 had both constipation and diarrhea manifesting as alternating episodes, 6 had only diarrhea, and 15 had only constipation

^b^ In most patients, it was not possible to definitely localize the pain to the upper or lower abdomen, due to their limited ability to communicate

^c^CT scan showed changes indicative of constipation

### Diagnoses, procedures and interventions (Tables 1 and 2)

Sixteen of the 41 patients (39%) had at least one procedure performed to aid in confirming the diagnosis (Mean: 1.87 + 1.09, Median: 2, Range: 1–5 per patient). Table 1 shows the procedures performed with the numbers and percentages of abnormal results for each procedure. Table 2 shows the various diagnoses and types of interventions. Signs and symptoms indicative of gastrointestinal dysmotility were seen in 33 patients (80%). These included esophageal phase dysmotility, abdominal distension, diarrhea, and constipation. Oropharyngeal dysphagia was seen in 26 patients (63%). Often episodes of constipation or even pseudo-obstruction led to anorexia vomiting and dehydration. This was often associated with precipitation of hemiplegia, dystonia and/or epileptic seizures in the short term. Increaseed incidence of these spells was usually also followed by exacerbation of the gastrointestinal symptoms, leading to poor weight gain in the long term. All 41 patients required medical attention including advice about feeding to address swallowing problems, and/or medications, such as for reflux or constipation, and/or surgery (Table 2). The combination of motility problems and dysphagia led to G-tube placement in 16 (39%). Gastroesophageal reflux which occurred in 26 (63%) required fundoplication in 2 (4.9%) after conservative medical therapy failed. Lastly, 4 patients had a confirmed diagnosis of gastroparesis via a nuclear medicine gastric emptying study.

**Table 2:** Gastrointestinal Diagnoses and corresponding interventions in our cohort

Diagnosis	Number affected/Number needing medical intervention/number needing surgery
Gastrointestinal dysmotility	33 affected/33 needed medications/16 needed gastrostomy^a^
Gastroparesis	4 affected/4 diagnosed by nuclear medicine gastric emptying study, 0 needed surgery
Oropharyngeal Dysphagia	26 affected/26 needed advice about volumes and textures of food/16 needed gastrostomy^a^
Gastroesophageal Reflux	26 affected/26 needed medications/2 needed Nissen fundoplication

^a^Gastrostomy was performed for the problems of swallowing with aspiration as well as for usually coexisting hypomotlity

### Correlations with paroxysmal and non-paroxysmal AHC disease manifestations (Table 3)

The severity of the gastrointestinal symptoms correlated with the non-paroxysmal disability index and GMFCS scores but not with the paroxysmal disability score. In addition, concordance between gastrointestinal manifestations and non-gastrointestinal autonomic dysfunction was statistically significant, with a Cramer’s V of 0.361 and a *p*-value of 0.0166: 38 patients had both gastrointestinal symptoms and non-gastrointestinal type autonomic spells, 3 had the former without the latter, 2 had the spells without gastrointestinal symptoms and 1 had neither.

**Table 3:** Correlations between gastrointestinal symptom severity scores with disease severity scores (Spearman’s correlation) and with presence or absence of non-gastrointestinal autonomic symptoms (Cramer’s V test)

	Correlation Coefficient/ Cramer V values	*P*-Value
Paroxysmal Scores	−.128	.408
Non-paroxysmal Scores	.325	.031
GMFCS	.307	.043
Autonomic dysfunction spells presence	.361	.016

## Discussion

Our study demonstrates that gastrointestinal problems are very common in AHC and are predominantly related to gastrointestinal dysmotility and dysphagia.

### Symptoms

We found that gastrointestinal symptoms are a common cause of morbidity in AHC, occurring in 93% of patients. This is not surprising since such problems are also common in other disorders affecting the central nervous system, such as cerebral palsy [13]. In that study, no finding on neuroimaging had a significant relationship to the presence of any gastrointestinal symptom. This suggested that many of these symptoms in cerebral palsy might not be related to specific brain lesions, but to a disrupted central modulation of gut activity. In our study, only a minority of our patients had abnormal MRIs, indicating that the occurrence of gastrointestinal dysfunction in AHC is not dependent on the presence of specific MRI detectable brain lesions. Although medications likely contributed to the gastrointestinal symptoms, the number of patients taking each class of medications, and the overlap of the medication regimens did not allow for statistical testing of the possible associations of gastrointestinal symptoms with medication intake.

### Diagnoses and interventions

We found that gastrointestinal dysmotility problems were diagnosed 75% of our AHC patients and 80% of those with gastrointestinal symptoms. Motility disorders are common in several neurological disorders such as Rett syndrome and mitochondrial disorders, including the mitochondrial neuro-gastrointestinal encephalopathy (MINGIE) syndrome [14, 15]. In these disorders, and as we found in AHC patients, interventions are usually needed, with surgical interventions having been required in slightly over 1/3 of our patients. Physicians taking care of patients with AHC should be aware of motility and other gastrointestinal problems that can affect patients with AHC, and should have a low threshold to investigate for these disorders if suggestive symptoms occur. The occurence of neuropsychological impairments, severe hemiplegia, epileptic seizures, status epilepticus and dystonia spells often overshadows the gastrointestinal manifestations and monopolizes neurologists’ and primary care physicians’ time in the care of AHC patients [16]. This emphasizes the importance of multidisciplinary team care of those patients. As compared to the European cohort reported by Panagiotakaki et al. in 2010 [11], the median paroxysmal disability index score divided by the numbers of variables assessed was around 2.5 in that study and it was 2.33 in our study. Similarly, the median nonparoxysmal disability index score in that study was in the 1–1.25 range, while in our study, it was 0.71 [11]. This indicates a similar, if not lower, severity of symptoms in our series as compared to that series. In addition, in the Japanese study of Sasaki et al., about a quarter of their patients were noted to have severe catastrophic regression [16]. This was noted in only one of our patients. Anticipatory guidance and addressing the above gastrointestinal disorders is important since early diagnosis and management can make a difference. Specifically, failure to gain weight, diarrhea, vomiting, dehydration, and constipation can make AHC children more vulnerable not only to intercurrent infections and illnesses, but also to worsening of their AHC symptoms. This includes the triggering of severe prolonged hemiplegias, quadriplegias, dystonias, epileptic seizures and status epilepticus that can lead to catastrophic and irreversible regression in their neurological status [17, 18]. It can also lead to a vicious cycle in which gastrointestinal symptoms precipitate AHC neurological complications, and these complications in turn exacerbate the gastrointestinal symptoms. We have been able to avoid total parenteral nutrition in our patients through the use of G-tube feedings and with anticipatory guidance. However, we are aware that one of our patients who transferred care to another facility did need total parenteral nutrition after the move. Furthermore, we recently saw another patient at our medical center, who is not included in this cohort of consecutive patients because he was seen after we performed the analysis of our current cohort, who had been started on total parenteral nutrition by prior providers. Thus, a more proactive approach and anticipatory guidance may potentially limit exacerbations in gastrointestinal symptoms, and reduce the need for surgical interventions, as well as for total parenteral nutrition.

### Correlations with other AHC symptoms and with known pathophysiology of AHC and gastrointestinal disease

We found that there was a positive correlation between the severity of the non-paroxysmal disease severity scales of AHC and the severity of the gastrointestinal symptoms. This should be useful in counseling families, in delivering follow up care, and in anticipatory guidance.

The mechanisms of gastrointestinal symptoms in AHC may be non-specific, related to general neurological dysfunction such as is seen in cerebral palsy. On the other hand, they may be, at least in part, related to the specific underlying AHC pathophysiology. Recent investigations have revealed high concentrations of the α3 Na, K-ATPase subunit in all hypothalamic nuclei [8, 9]. The hypothalamus projects to the lateral medulla and regulates sympathetic and parasympathetic pathway functions [19]. These pathways control gastrointestinal function and motility. Also, the hypothalamic arcuate nucleus is known to regulate feeding behavior and appetite [20]. The α3 subunit is also expressed in all vagal afferent neurons [21]. The spinal trigeminal motor nucleus, which integrates sensory input from the dental and craniofacial regions and feeds into the hypothalamus, modulates vagal responses and is strongly positive for α3 Na, K-ATPase, at least in mice fetuses [9, 22]. Additionally, even though there is no evidence of α3 Na, K-ATPase expression in any non-myelinated peripheral nerves of the autonomic nervous system [23], the α3 Na, K-ATPase is distributed in dorsal root ganglia neurons [23–25], which may potentially affect reflex function in the gastrointestinal tract.

The α3 Na, K-ATPase is highly expressed in motor brainstem nuclei important in oropharyngeal function, which likely explains the difficulty AHC patients have in swallowing [7, 10]. This also likely explains why other disorders caused by ATP1A3 mutations, such as rapid onset dystonia parkinsonism (RDP) and relapsing encephalopathy with cerebellar ataxia (RECA) manifest marked bulbar findings of dysarthria, hypophonia, and dysphagia [10, 26]. Finally, it is important to note that GABAergic dysfunction has recently emerged as an important mechanism affecting gastrointestinal motility [27]. Knock-in mice carrying the D801N mutation, the most common mutation causing AHC in humans, demonstrate marked GABAergic interneuron dysfunction [28]. Furthermore, many other central nervous system disorders that manifest gastrointestinal motility problems, including mitochondrial disease and Rett syndrome, also have been shown to manifest prominent GABAergic dysfunction [29]. Lastly, cerebellar function contributes to gastrointestingal motility, and cerebellar dysfuntion is very common in AHC [4–7, 30–32].

### Potential limitations, advantages, and future directions

All our patients fit the Aicardi AHC clinical diagnostic criteria. Although *ATP1A3* mutations can cause other phenotypes, we specifically focused on AHC and did not study patients with other *ATP1A3* related phenotypes [26]. We suggest that this would be an interesting subject for future investigations. Formal motility testing was conducted on only some of our patients. However, this is the case because our study reflects results based on actual clinical care practices. Also, patients in this study may have been selectively referred to our center because of their disease severity and the availability of our multidisciplinary clinic. Finally, demonstration of an association with neurological symptoms does not necessarily imply causality. Nonetheless, the neurological manifestations of the patient population we see in our center are comparable to other series previously described in Europe, USA and China [4–7, 17, 33] and appear to be less severe than the patient population reported from Japan [16]. Additionally, although this study is not a longitudinal study, it has the advantage of a hypothesis-driven comprehensive analysis of the available gastrointestinal-related manifestations and data of an informative cohort of consecutive AHC patients. Future studies with larger numbers of patients could investigate if such larger numbers could delineate subtle differences that may exist between the mutation positive and mutation negative patients that the numbers of patients we had may not have detected.

## Conclusions

Gastrointestinal problems are common and are at times severe in patients with AHC. Awareness of these issues and anticipatory guidance should help in planning the correct medical and surgical interventions. Our findings are consistent with current understanding of the underlying pathophysiology of AHC that involves ATPase related neuronal dysfunction in regions that control gastrointestinal function/motility and swallowing.

## Data Availability

The datasets used and/or analyzed during the current study are available from the corresponding author on reasonable request.
